# Hyperalphalipoproteinemia and Beyond: The Role of HDL in Cardiovascular Diseases

**DOI:** 10.3390/life11060581

**Published:** 2021-06-18

**Authors:** Antonina Giammanco, Davide Noto, Carlo Maria Barbagallo, Emilio Nardi, Rosalia Caldarella, Marcello Ciaccio, Maurizio Rocco Averna, Angelo Baldassare Cefalù

**Affiliations:** 1 Department of Health Promotion, Mother and Child Care, Internal Medicine and Medical Specialties–University of Palermo, Via del Vespro, 129, 90127 Palermo, Italy; agiamman@gmail.com (A.G.); notoddd@gmail.com (D.N.); carlo.barbagallo@unipa.it (C.M.B.); emilio.nardi@unipa.it (E.N.); maurizio.averna@unipa.it (M.R.A.); 2 Department of Laboratory Medicine, Unit of Laboratory Medicine CoreLab, University Hospital “P. Giaccone”, 90127 Palermo, Italy; rosalia.caldarella@policlinico.pa.it (R.C.); marcello.ciaccio@unipa.it (M.C.); 3 Department of Biomedicine, Neurosciences and Advanced Diagnostics, University of Palermo, 90127 Palermo, Italy

**Keywords:** hyperalphalipoproteinemia, HDL, CETP, polymorphisms, cardiovascular disease

## Abstract

Hyperalphalipoproteinemia (HALP) is a lipid disorder characterized by elevated plasma high-density lipoprotein cholesterol (HDL-C) levels above the 90th percentile of the distribution of HDL-C values in the general population. Secondary non-genetic factors such as drugs, pregnancy, alcohol intake, and liver diseases might induce HDL increases. Primary forms of HALP are caused by mutations in the genes coding for cholesteryl ester transfer protein (CETP), hepatic lipase (HL), apolipoprotein C-III (apo C-III), scavenger receptor class B type I (SR-BI) and endothelial lipase (EL). However, in the last decades, genome-wide association studies (GWAS) have also suggested a polygenic inheritance of hyperalphalipoproteinemia. Epidemiological studies have suggested that HDL-C is inversely correlated with cardiovascular (CV) risk, but recent Mendelian randomization data have shown a lack of atheroprotective causal effects of HDL-C. This review will focus on primary forms of HALP, the role of polygenic inheritance on HDL-C, associated risk for cardiovascular diseases and possible treatment options.

## 1. Introduction

Hyperalphalipoproteinemia (HALP) is a condition characterized by elevated plasma high-density lipoprotein cholesterol (HDL-C) levels > 90th percentile of the distribution of HDL-C values in the general population [1], associated or not with overt clinical manifestations and predisposition to atherosclerotic coronary artery disease (CAD) [2]. Plasma total cholesterol (TC) levels may be increased, while very low-density lipoproteins (VLDL) and low-density lipoproteins (LDL) are often in the normal range. HALP is classified as moderate (HDL-C levels between 80 and 100 mg/dl) and severe (HDL-C levels > 100 mg/dl) [2]. HALP is the hallmark of primary hyperalphalipoproteinemia, a heterogeneous genetic lipoprotein disorder, usually transmitted as a co-dominant trait, due to mutations in known candidate genes or to other genes yet to be identified (“orphan” primary HALP). Mutations in the genes coding for cholesteryl ester transfer protein (CETP), hepatic lipase (LIPC), and apolipoprotein C-III (APOC3) are known causes of primary HALP [1]. The pathophysiology of other forms of HALP is not well characterized, and it is still unknown if the increased production or reduced catabolism of HDL are the cause of this lipid disorder [1]. Epidemiological studies have demonstrated a strong inverse relationship between low HDL-C levels and risk for developing atherosclerotic cardiovascular disease (ASCVD) [3,4]. On the other hand, Mendelian randomization studies have failed in demonstrating a causal relationship between HDL-C and ASCVD [5]. Low plasma HDL-C levels strongly correlate with high CV risk, but genetically determined low HDL-C levels are not associated with an increased risk for ASCVD, suggesting that low HDL-C levels per se are not a cause of cardiovascular diseases [5,6]. Epidemiological studies have shown contradictory results on the relationship between high HDL levels and CV risk in subjects with primary HALP [7,8]. Several mechanisms may play a role in explaining this phenomenon, including HDL function in reverse cholesterol transport (RCT) [9]. Besides the major involvement in RCT, HDLs exert several anti-inflammatory effects that may prevent endothelial dysfunction [10], which is considered one of the first events in atherogenesis [11].

## 2. HDLs Physiology

**Figure 1:**
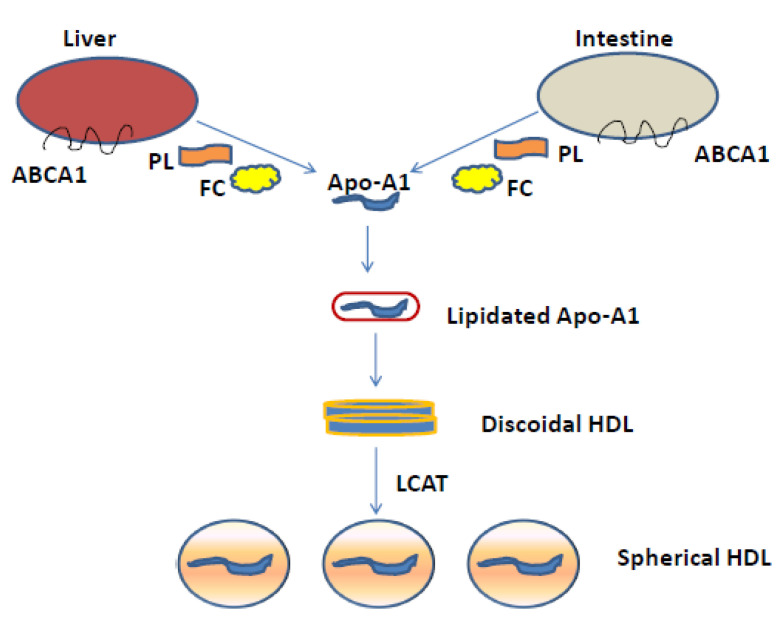
Schematic representation of the biogenesis of HDL. The first step in HDL biogenesis begins with the secretion of Apo-A1 by the liver and the intestine. The lipid-poor Apo-A1 then interacts with ABCA1 and progressively gains phospholipids (PL) and free cholesterol (FC) from the cells. The lipidated apoA-I is gradually converted to discoidal particles composed of unesterified cholesterol. Then, the enzyme lecithin/cholesterol acyltransferase (LCAT) esterifies the FC and the discoidal HDLs are finally converted to spherical HDL particles containing Apo-A1, Apo-E or Apo-A4: secondary causes of hyperalphalipoproteinemia.

**Figure 2:**
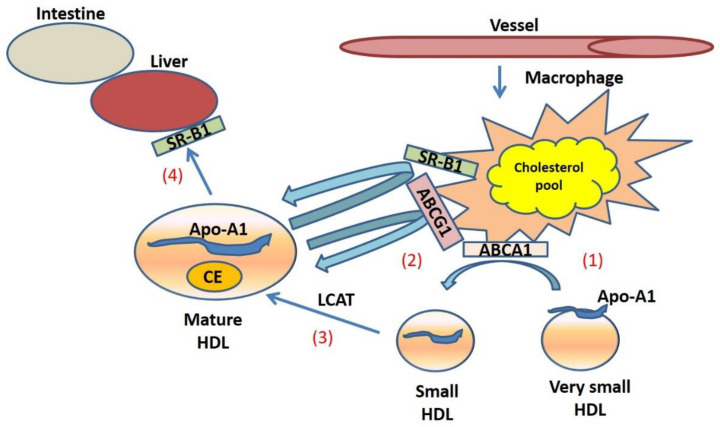
Schematic representation of the Reverse Cholesterol Transport (RCT) mechanism. In the RCT process, the ApoA-I receives the cholesterol from the foam cells (macrophages) through the ATP-binding cassette transporter member 1 (ABCA1) (**1**), leading HDL to become mature in a mechanism mediated by the transporter ABCG1 (**2**). The Lecitin:cholesterol acyltransferase (LCAT) (**3**) esterifies the free cholesterol (FC) thus contributing to form mature and spherical HDLs formed of a cholesteryl ester (CE) core. Finally, HDLs bind to the scavenger receptor class B type-1 (SR-B1) in the liver (**4**) and are selectively removed from the blood stream.

HDLs are characterized by a heterogenous sub-population of lipoprotein particles, which undergo remodeling and transformation processes mediated by several plasma enzymes and transcription factors [12]. The main HDL-associated apolipoprotein is apolipoprotein AI (apoA-I), a protein of 243 amino acids [13] which is synthesized by the liver and intestine and represents the principal structural component of HDLs. Schematic representation of the biogenesis of HDL is illustrated in Figure 1. HDL particles are involved in the so-called “reverse cholesterol transport (RCT)”, a pivotal pathway involved in the return of excess cholesterol from peripheral tissues to the liver for excretion in the bile and eventually in the feces. RCT from macrophages in atherosclerotic plaques (macrophage RCT) is a critical mechanism of the antiatherogenicity of high-density lipoproteins (HDLs) (Figure 2) [14,15,16,17,18,19,20]. Besides their major role in promoting cell cholesterol efflux and reverse cholesterol transport, HDLs may exert atheroprotective activity by preventing endothelial dysfunction [10], a key step in the development of atherosclerosis. HDL downregulates cytokine-induced expression of cell adhesion molecules (CAMs) [10] and increases endothelial nitric oxide synthase (eNOS) expression and activation [21], NO release, and bioavailability [22]. HDLs induce the production of NO by increasing the endothelial nitric oxide synthase (eNOS) activity, thus improving the endothelial function [23,24]. Besides the antioxidative properties of apoA-I, the HDL accessory protein—paraoxonase 1 (PON1)—may exert an important role in determining the antioxidative capacity of HDL particles, and is implicated in reverse cholesterol transport and atheroprotective effects [25]. In addition, HDLs together with ABCA1 and ABCG1 may play an anti-atherogenic role by inhibiting hematopoietic stem cell (HSC) proliferation and suppressing macrophage activation, thus decreasing inflammatory responses [26].

Although in this review we will mainly focus on the primary familial causes of HALP, it is worth mentioning that several conditions are known to be associated with elevated HDL-C levels. Patients with secondary HALP tend to be asymptomatic, aside from some rare reported cases of juvenile or premature corneal opacities [27] or multiple symmetric lipomatosis [28], and are characterized by high levels of HDL-C and a low incidence of CVD [29]. Lifestyle factors include vigorous and sustained aerobic exercise, regular and substantial alcohol consumption, and weight loss, and the increase in HDL-C levels may be mainly attributed to CETP and/or HL inhibition [30,31,32]. HDL-C levels are known to be higher in women than in men, and estrogens play an important role in this phenomenon [30,33]. In users of combination oral contraceptives, the rising effect of estrogen is partly counter-regulated by the presence of added progestin such levonorgestrel, which exerts an androgenic effect by decreasing the Apo-AI synthesis and increasing LPL activity [34]. In uncomplicated pregnancy, especially over the second and third trimester, women may exhibit an increase in HDL-C levels due to a reduction in CETP activity (33). The liver is one of the main sites for HDL catabolism [35], and in some chronic conditions, such as biliary cirrhosis, HDLs may accumulate in the bloodstream because of a defect in their catabolism giving rise to secondary HALP [35]. Several classes of commonly used drugs in clinical practice exert effects both on HDL levels and functions through various mechanisms. Among them are anti-inflammatory medication (NSAIDs, corticosteroids, methotrexate, sulfasalazine, hydroxychloroquine or biologics) [36,37,38] and lipid-lowering drugs (statins, fibrates, and niacin) [39].

## 3. Primary Causes of HALP

### 3.1. CETP Deficiency

The CETP gene maps on the long arm of chromosome 16 (16q12-16q21); it comprises 16 exons [40] and encodes for a glycosilated plasma protein which catalyze the exchange of triglycerides (TGs) and cholesteryl esters (CEs) among lipoprotein particle cores [41,42]. In more detail, it mediates the transport of CE from HDL to VLDL/chylomicrons and TG from VLDL/chylomicrons to HDL and low-density lipoproteins (LDL) [41,43], thus regulating HDL-C plasma levels. The genetic epidemiology of HALP is mostly based on studies performed in Japan, the country with the highest known prevalence of primary HALP and where CETP deficiency was first described in 1985 [44,45,46]. Since then, several cases of CETP deficiency due to mutations of the CETP gene have been reported in the Japanese population, and it has been shown that in subjects with moderate and severe HALP, the prevalence of CETP deficiency is ~60% and ~31%, respectively [47,48]. According to Japanese epidemiological data, 27.6% of Japanese subjects with HDL cholesterol > 60 mg/dl, and 31.4–32.5% of those with HDL-cholesterol > 80 mg/dl are carriers of CETP gene mutations [49]. Three CETP gene mutations account for most cases among the Japanese: intron 14 G (+1) > A (Int14A) splicing defect (1–2%), exon 15 missense mutation (D442G) (14) (6–7%) and the nonsense mutation G309X [50,51]. CETP deficiency is quite common in other Asian populations (mostly Chinese, Siberians and Thai) [52], and with this observation a founders’ effect might be hypothesized [52]. However, this genetic defect is rare in other ethnic groups, even though some studies have reported sporadic cases of CETP deficiency in the United States [53], Italy, Greece and the Netherlands [54,55,56,57]. Resequencing of the CETP gene in White Americans and Black Africans allowed the identification of rare variants (allele frequency < 0.01), which may explain the extremely high HDL-C phenotype in both groups [58]. The loss-of-function CETP protein results in significantly elevated HDL-C levels in homozygotes (usually > 100 mg/dL), in whom CETP mass and/or activity is not detectable in plasma [33,47] and moderately elevated HDL-C levels in heterozygotes with a CETP mass half of that compared to healthy controls [33,47]. The lack of CETP activity is responsible for the accumulation of CE in HDLs that becomes larger (>11 nm); on the other hand, LDL-C levels tend to be low and LDL particles are rich in triglycerides and polydisperse with a subpopulation of small LDLs [1,2,45,48,59,60]. Moreover, in CETP-deficient subjects, HDL size correlates inversely with the CETP mass and activity, and HDLs are larger not only in normal subjects but also in patients with other forms of HALP [61]. This key biochemical feature in CETP deficiency influences HDL functional properties and may help to discriminate the CETP deficiency from other genetic causes of HALP [62,63]. In addition, the LDL particles in CETP deficiency [60] are compositionally altered and display a low affinity for LDL receptor and may be atherogenic [60]. Plasma apolipoproteins AI, CIII and E are increased while apoB is normal or decreased [63]. Even though CETP deficiency is associated with high HDL-C and decreased LDL-C, its role in atherosclerotic cardiovascular diseases has been controversial. In an early observation by Inazu et al., 10 subjects with very HDL-C levels due to complete CETP deficiency, belonging to five unrelated families, did not have any premature atherosclerotic diseases, and two families displayed a trend toward longevity [64]. In the Honolulu Heart Program, 3469 Japanese male subjects, carriers of two different CETP gene mutations, were evaluated for correlations between CETP deficiency, HDL-C levels and cardiovascular diseases [7]. It was found that male heterozygous carriers of CETP gene mutations with low or slightly increased HDL-C levels (1.0–1.6 mmol/L) exhibited a higher cardiovascular risk than non-carriers matched for gender and HDL-C levels [7]. On the other hand, males with considerably elevated HDL-C levels (>1.6 mmol/L), regardless of the CETP gene status, had a low frequency of cardiovascular disease (coronary heart disease) [7]. Furthermore, analysis of cardiovascular outcomes in a Japanese cohort of 19,044 males and 29,487 females showed that subjects with both markedly elevated and mild-to-moderate HDL-C levels experienced fewer cardiovascular diseases independently of the status of the CETP gene mutation carrier [65]. To date, the correlation between CETP and cardiovascular diseases is still controversial, as is its role on longer life expectancy. Further studies are needed to clarify these observations [66,67].

### 3.2. Hepatic Lipase and APO-CIII Deficiency

HL deficiency is another cause of monogenic HALP. Hepatic lipase (HL) is a heparan-sulfate proteoglycan (HSPG)-bound lipolytic enzyme synthesized and secreted by hepatocytes. It is encoded by the LIPC gene mapping on chromosome 15 [68], and it is involved in HDL and triglyceride metabolism [69,70,71,72,73,74]. Single-nucleotide polymorphisms (SNPs) of LIPC may be pro-atherogenic, whereas others induce an anti-atherogenic phenotype [69,75,76]. This different role of LIPC SNPs is influenced by secondary influences such as environmental, lifestyle and hormonal factors [77]. HL deficiency may increase the HDL size and their functions in the RCT process, thus increasing the risk of premature cardiovascular diseases [72]. To date, only a few case reports of families with primary HALP caused by a genetically defined HL deficiency have been described [78,79,80,81,82]. Polymorphisms in the HL gene promoter act as modifiers of HDL-C levels, but the moderate increase in HDL induced by these polymorphisms cannot explain the high HDL levels observed in primary HALP. **APO-CIII.** Apolipoprotein CIII (Apo-CIII) is a small apolipoprotein synthesized mainly in the liver and regulates plasma TG homeostasis by inhibiting lipoprotein lipase (LPL) activity [83]. Apo-CIII plays an important role in HDL metabolism as well as in TG physiology. Loss of function of APO-CIII gene mutation carriers exhibits 39% lower plasma TG levels, 22% higher plasma HDL-C levels, 16% lower plasma LDL-C levels, and reduction in CVD risk [84,85]. Two novel loss-of-function mutations which affect the splice site of the **APOC3** gene (c.13-2A > G and c.55+1G > A) have been identified and associated with plasma HDL-C levels above the 95th percentile and an atheroprotective lipid profile [86]. A missense APOCIII variant (Lys58 > Glu) in heterozygosity was described in two women with hyperalphalipoproteinemia [87].

### 3.3. Scavenger Receptor Class B Type I (SR-BI)

The scavenger receptor class B type I (SR-BI), encoded by the SCARB1 gene, is primarily expressed in the steroidogenic tissues and in the liver, where it acts as an important receptor for HDLs and controls selective uptake of the cholesterol esters by HDL [88]. SR-BI is involved in the bi-directional transfer of esterified cholesterol between cells and HDL [89]. SR-BI knock-out mice exhibited a twofold increase in HDL-C plasma levels, accelerated atherosclerosis, impairment of liver cholesterol transfer [90], and adrenal glucocorticoid-mediated stress response [91]. SCARB1 rare point mutations associated with a decreased SR-BI protein expression and function have been identified in subjects with high plasma HDL-C levels in humans [92,93,94]. However, despite the high plasma HDL-C concentration, carriers exhibit an increased risk of CVD due to the impaired RCT pathway caused by the reduced hepatic SR-BI function [95,96,97]. Carriers of the P376L mutation have been shown to have a 79% higher risk of CHD, as compared to non-carriers. Vergeer M. et al. re-sequenced SCARB1 genes in subjects with high HDL-C levels (>70.4 mg/dl to 1.8 mmol/L) and identified a family carrying the missense mutation P297S, which co-segregated with high HDL-C levels, decreased the cholesterol efflux from macrophages, increased platelet dysfunction, and reduced adrenal steroidogenesis [94], although without significant impact on atherosclerosis [94]. Yang X. et al. have described the association of SCARB1 variants resulting in a decreased function of SR-B1 in the binding/intracellular transport of Lp(a) associated with a peculiar, combined lipid phenotype characterized by elevated HDL-C and Lp(a) levels [98].

### 3.4. Endothelial Lipase (EL)

Endothelial lipase (EL) is mostly involved in HDL phospholipid hydrolysis [99] through a mechanism independent from the dissociation of lipid-free/lipid-poor apoA-I [100]. EL is coded by the LIPG gene and it is expressed mainly in endothelial cells [99], but also in other several tissues including the liver, lungs, placenta, thyroid, kidney, and macrophages [99]. EL, together with hepatic lipase (HL), exerts a negative regulation in HDL metabolism and modulates the cholesterol efflux capacity (CEC) of serum and isolated HDL [101,102]. Experiments in EL and HL knock-out mice have shown that these two lipases affect the RCT process and promote the HDL antioxidant properties [102]. Carriers of loss-of-function variants of EL have a reduced lipolytic activity [103] and a lipid phenotype characterized by an increase in HDL-C plasma levels with large HDL particles [100,101,104]. Further studies are needed to evaluate the role of loss-of-function variants and if reduced EL activity might be cardioprotective [105].

### 3.5. Polygenic Causes of Hyperalphalipoproteinemia

Several polymorphisms of the CETP gene with a reduction in CETP activity have been associated with HDL-C levels in the general population [106]. TaqIB is a silent base change affecting a nucleotide at position 277 on the first intron of the CETP gene and represents a common polymorphism associated to increased HDL cholesterol plasma levels and to a slight reduction in cardiovascular risk [107]. Some CETP genotypes correlate with a mild reduction in CETP activity, a marginal HDL-C increase, and an inverse association with coronary artery disease [108]. The variation in CETP activity is much better explained by using a haplotype model consisting of TaqIB and four other polymorphisms in the CETP gene [109]. Different studies (the Framingham Offspring Study, the Veterans Affair HDL-c Intervention Trial and the WOSCOPS) have confirmed the association of CETP and a low CV risk in males [110,111,112], as has the AtheroGene Study, which, by evaluating 1211 patients with coronary artery disease (CAD) on follow-up, has demonstrated an association of the A allele of the CETP-629 variant with a reduced CV lethality [113]. Another haplotype analysis demonstrated a correlation between the -2505 CETP variant and HDL metabolism and CV risk [114]. Interestingly, a study performed in southern China (Hainan) has shown a correlation between CETP polymorphisms and longevity: TaqIB and the variant I405V were combined and analyzed in a group of centenarians matched with controls [115], and it was found that the alleles B1 and V contributed to a protective role on longevity in this cohort of subjects. In the last decade, genome-wide association studies (GWAS) have suggested a more complex polygenic inheritance of HDL plasma levels [116]. Beside the known candidate genes of primary HALP, other genes (such APOA1, LCAT, APOA4, APOE, PLTP and PON1) involved in HDL metabolism have been reported to modulate HDL-C plasma levels [117,118,119]. Although several genes have been discovered to be associated with HDL metabolism, to date, only a small percentage of this genetic variability can be explained and their effects on HDL phenotypes should be further investigated and probably related to environmental factors [120,121]. Further genetic variants will be identified; therefore, it will be possible to predict, through an allelic risk score, subjects at high cardiovascular risk and possible strategies for prevention [122].

### 3.6. HALP and Cardiovascular Risk

Following the seminal data provided by Gofman J.W. et al. [123] and the Framingham study [124], several studies have confirmed the inverse relationship between HDL-C plasma levels and coronary heart disease (CHD), suggesting an atheroprotective role of HDLs [125,126,127]. Furthermore, some studies have reported a positive correlation between elevated HDL-C and longevity [128]. The Long-Life Family Study (LLFS) has demonstrated that subjects with high HDL-C are healthier in terms of CV outcomes [129]; a study on aging male veterans revealed that elevated HDL-C correlates with longer life expectancy [128]. However, recent findings provided by Mendelian randomization studies support the hypothesis that some genetic mechanisms that raise plasma HDL cholesterol do not seem to lower risk of myocardial infarction [5,6]. These data may question the concept that HALP and/or the pharmacological raising of plasma HDL cholesterol will translate into atheroprotection and a reduction in risk of myocardial infarction [130]. For example, in statin clinical trials, ApoAI was inversely related to low CV risk, whereas HDL-C was not [131]. In addition, in the JUPITER trial of treatment with the high-intensity statin rosuvastatin, HDL-C was not a predictive factor of residual CV risk [132]. In LDL receptor KO mice, naturally lacking CETP, the expression and overexpression of CETP led to an atherogenic phenotype [133]. On the other hand, inhibition of CETP activity in rabbits leads to a condition mimicking the human primary HALP due to CETP deficiency with a striking HDL increase (about 90%) and antiatherogenic effects. Targeting the SR-B1 gene in mice increases HDL-C levels but is associated with accelerated atherosclerosis [133]. In brief, in animal models, the targeting of different HDL metabolism-related genes produces the same HDL-C increase but may induce atherogenic or atheroprotective effects based on the metabolic scenario and on the role that the different genes play in the specific biochemical pathway. In humans, HALP due to CETP deficiency represents a unique setting in which the high HDL-C and cardiovascular disease relationship has been studied. In Japan, CETP-deficient patients with HDL-C levels > 80 mg/dl do not seem to be protected against atherosclerotic vascular disease [7]. A recent hypothesis raised the focus on a dysfunctional RCT process which might increase the plasma HDL-free cholesterol (HDL-FC) level [16]. Thus, HDL-FC levels may represent a valuable biomarker independent of HDL-cholesteryl ester (HDL-CE and TC to assess cardiovascular risk, progression of ASCVD and response to lipid lowering treatments). HALP subjects exhibit higher levels of HDL-FC, and the plasma efflux capacity is lower than in those with normal HDL-C [16]. This process, which regulates FC bioavailability, could exert potentially toxic and pro-atherogenic properties on several tissues, including the artery wall, thus increasing the risk of atherosclerosis [16]. The RCT model was supported by several large studies which have revealed an inverse correlation between macrophage cholesterol efflux to plasma HDL and ASCVD [18]. In the Copenhagen City Heart Study and in the Copenhagen General Population Study, two large prospective population-based studies, a U-shaped association between HDL plasma levels and overall and cardiovascular mortality has been observed in both males and females, with a more pronounced risk of all-cause mortality in men [134,135]. The lowest frequency of CV events was observed for HDL-C levels close to 58 mg/dl (1.5 mmol/L) for men and 77 mg/dl (2.0 mmol/L) for women, and no further CV protection was evidenced with HDL-C plasma levels higher than these cut-offs [134]. Moreover, in the Copenhagen City Heart Study, it was demonstrated that two CETP polymorphisms were correlated with a reduced cardiovascular risk as well as longevity [136]. Similarly, in the CANHEART (Cardiovascular Health in Ambulatory Care Research Team) study [137] and in the study by Bowe et al. [138], increases in CV disease at HDL-C levels > 90 mg/dl (2.3 mmol/L) and > 90th percentile, respectively, were associated with increased hazard risk for mortality [138]. In summary, low HDL-C remains a significant factor for increased disease risk, whereas high HDL-C levels are not associated with cardioprotection, and this should prompt a re-evaluation of high HDL-C cutoffs in CVD risk calculations [136,137]. It has been hypothesized that this paradox could be the result of larger and dysfunctional HDL particles that might remain entrapped in the arterial intima, thus promoting cholesterol deposition and atherosclerosis progression in subjects with extremely high HDL-C levels [139].

### 3.7. Pharmacological Targets to Increase HDL-C

The concept that targeting HDL-C may be advantageous in terms of CV risk reduction has been taken into consideration for decades as an important treatment strategy. Niacin is a potent HDL-C-raising drug, seemingly an attractive approach to reduce cardiac events in patients with or at risk of atherosclerotic cardiovascular disease [140]. However, over the years, several clinical trials have failed to demonstrate benefits in terms of cardiovascular endpoints [141,142,143,144,145]. Table 1 shows a schematic view of the major niacin-based trials and their effects on HDL-C and on CV outcomes [144,145,146,147,148,149,150,151,152,153]. Fibrates have been used over the years to increase HDL-C, and their effects were evaluated in several clinical trials (the most representative are the FIELD—Fenofibrate Intervention and Event Lowering in Diabetes; ACCORD—Action to Control Cardiovascular Risk in Diabetes; VA-HIT—Veterans Administration HDL Intervention Trial, and the HHS—Helsinki Heart Study), although have failed in showing a significant CV risk decrease despite an HDL-C increase [142]. Statins have shown a consistent effect on HDL-C increases, and several trials have demonstrated this property. Among these, the VOYAGER study has evaluated the role of rosuvastatin (5–40 mg), atorvastatin (10–80 mg) and simvastatin (10–80 mg) in HDL-C increases, which were elevated as well associated with LDL-C decreases [154].

## 4. CETP Inhibitors

CETP inhibitors have been proposed over the years to raise HDL-C plasma levels, although clinical trials have failed to prove their hypothesized positive effects [155,156]. Among these, torcetrapib was the first CETP inhibitor developed to treat hypercholesterolemia and prevent atherosclerotic cardiovascular diseases. Although torcetrapib was effective in raising HDL-C and ApoA-I and reducing LDL-C with and without an added statin [156], phase 3 trials failed to demonstrate effects on atherosclerosis burden and cardiovascular deaths (RADIANCE and ILLUMINATE trials) [157,158]. Moreover, because of an excess of overall mortality and cardiovascular events, the development of torcetrapib was halted [156]. The excess deaths and adverse cardiovascular events in patients taking torcetrapib have been attributed to off-target effects independent from CETP inhibition [156,159]. Other potent and selective inhibitors of CETP have been developed and tested in clinical trials (such as dalcetrapib, anacetrapib, and evacetrapib). Although these drugs were effective in increasing HDL-C and reducing LDL-C without torcetrapib-like off-target liabilities, they failed to impact cardiovascular risk and outcomes [160,161,162]. Moreover, meta-analysis based on clinical trials carried out with niacin, statins, fibrates and CETP inhibitors have exhibited no decreases in CV mortality [160,163].

## 5. Future Directions

Clinical trials are investigating the role of new targets to evaluate cardioprotection by increasing specific HDL-C subclasses or improving HDL functions [164]. Reconstituted HDL (rHDL) therapies, including an HDL mimetic molecule (CER-001), an apoAI derivative (CSL112), and a recombinant human LCAT (ACP-501) together with the antibodies anti-apoAI (anti-ApoAI IgG) could represent the basis for the development of targets focused on HDL3 sub-class (functionally superior to HDL2) increases, or based on the HDL functions, improvements rather than necessarily increasing HDL-C which has already been tested without any encouraging results [161,162].

## 6. Conclusions

In summary, primary HALP is a heterogenous genetic lipid disorder and some pathogenic mechanisms are still not completely understood. The supposed protective role of HDL on CV risk have been extensively evaluated over the years, and several studies have shown that different mechanisms based on HDL-C increases by enhancing both the cholesterol efflux and the cholesterol esterification are not definitely anti-atherogenic. Moreover, the different metabolic scenarios and specific biochemical pathways governed by the numerous HDL-related genes need further studies in order to clarify in which way a similar HDL-C increase may induce atherogenic or athero-protective effects. New functional assays would be necessary to measure the HDL quality in a validated, reproducible and cost-effective manner to better understand the mechanisms related to cardiovascular protection.

## Figures and Tables

**Table 1:** Major niacin-based trials and their effects on HDL-C and on CV risk.

Clinical Trial	Description	Effect on HDL-C and CV Outcomes	Reference
**AIM-HIGH**	3414 subjects were treated with niacin or placebo on top of high-intensity statin treatment	The niacin-treated arm showed a modest but significant HDL-C increase (25% vs. 12%) but no benefits in terms of cardiovascular outcomes. The trial was stopped earlier due to futility of results.	[144]
**HPS2-THRIVE**	25673 subjects affected by vascular diseases were randomized to receive extended-release niacin + laropiprant (to reduce the flushing side-effect of niacin) or placebo both on top of statin therapy	After a follow up mean of 3.9 year, the niacin-treated group showed a modest but significant HDL-C increase (6 mg/dl), but no difference in the incidence of CV events.	[145]
**Niacin Study Group**	Males with metabolic syndrome (obese, hypertriglyceridemic, non-diabetic) and low HDL-C levels received niacin for 8 weeks	A decrease in LDL-C and total cholesterol associated to a reduction in inflammation, cell-adhesion and proliferation biomarkers. No significant change of CV events.	[146]
**Coronary Drug Project (CDP)**	8 341 Males after myocardial infarction treated with niacin or clofibrate vs. placebo	HDL-C increase, LDL-C and TG decrease. No significant change of CV events.	[147]
**ARBITER-2**	167 patients with Coronary Artery Disease were treated with ER-niacin 1 g/day vs. placebo on top of stable statin therapy	HDL-C increase by 21%. Progression of cIMT in the niacin group	[148]
**ARBITER-6**	208 patients (≥30 years) with CAD or equivalent of CAD risk were treated with ER-niacin vs. ezetimibe on top of statin therapy	HDL-C increase in ER-Niacin group; reduced incidence of cardiovascular events by 5% in ER-Niacin group vs. 1% in EZE-group	[149]
**AFREGS**	143 patients (<76 years) with low HDL-C and coronary disease were treated with Niacin 0.25–3 g gemfibrozil 1.2 g cholestyramine 2 g vs. placebo	HDL-C increase by 36%; 13.7% decrease of combined cardiovascular events. No significant data.	[150]
**CLAS**	162 Males after CABG treated with Niacin 3–12 g/day + colestipol 30 g/day vs. placebo	HDL-C increase by 31%; TC decrease by 15–20% and LDL-C decrease by 43%; atherosclerotic regression in 16.2% of patients at 2 years and 17.9% at 4 years, compared with 2.4% and 6.4%, respectively, in the placebo group	[151]
**Stockholm trial**	558 patients after MI, aged <70 treated with Clofibrate 2×1 g + niacin 3×1 g vs. placebo	TC decrease by 26%; TG decrease by 30%; nonfatal Miocardial Iinfarction decrease by 50%	[152]
**HATS**	160 patients with CAD and low HDL-C distributed in 4 arms and treated with: Group A: simvastatin 10–20 mg/d plus niacin 2–4 g/d; Group B: antioxidant; Group C: simvastatin + niacin + antioxidant; Group D: placebo	LDL-C decrease by 42%; HDL-C increase by 26%; regression of severe coronary stenosis by 0.4% vs. placebo; 88% decrease of CV events (coronary death, MI or stroke, or revascularization)	[153]

AIM-HIGH (Atherothrombosis Intervention in Metabolic Syndrome with Low HDL/High Triglyceride and Impact on Global Health Outcomes); HPS2-THRIVE (Heart Protection Study 2—Treatment of HDL to Reduce the Incidence of Vascular Events); CDP (Coronary Drug Project); ARBITER-2 (Arterial Biology for the Investigation of the Treatment Effects of Reducing Cholesterol); ARBITER-6 (Arterial Biology for the Investigation of the Treatment Effects of Reducing Cholesterol-6-HDL); AFREGS (Armed Forces Regression Study); CLAS (The Cholesterol Lowering Atherosclerosis Study); HATS (HDL-Atherosclerosis Treatment Study). ER-niacin: extended release-niacin; cIMT: carotid intima-media thickness; CABG: coronary artery bypass graft surgery; TC: total cholesterol; TG: triglyceride; HDL-C: high-density lipoprotein cholesterol; LDL-C: low-density lipoprotein Cholesterol; CAD: coronary artery disease; MI: myocardial infarction.

## Data Availability

Data sharing not applicable. No new data were created or analyzed in this study. Data sharing is not applicable to this article.
